# Synthetic Microbiomes on the Rise—Application in Deciphering the Role of Microbes in Host Health and Disease

**DOI:** 10.3390/nu13114173

**Published:** 2021-11-21

**Authors:** Silvia Bolsega, André Bleich, Marijana Basic

**Affiliations:** Institute for Laboratory Animal Science, Hannover Medical School, 30625 Hannover, Germany; bolsega.silvia@mh-hannover.de (S.B.); bleich.andre@mh-hannover.de (A.B.)

**Keywords:** synthetic communities, minimal microbiota, intestinal microbiota, host–microbe interactions, gnotobiotic animal models, microbiome, intestinal diseases, metabolism

## Abstract

The intestinal microbiota conveys significant benefits to host physiology. Although multiple chronic disorders have been associated with alterations in the intestinal microbiota composition and function, it is still unclear whether these changes are a cause or a consequence. Hence, to translate microbiome research into clinical application, it is necessary to provide a proof of causality of host–microbiota interactions. This is hampered by the complexity of the gut microbiome and many confounding factors. The application of gnotobiotic animal models associated with synthetic communities allows us to address the cause–effect relationship between the host and intestinal microbiota by reducing the microbiome complexity on a manageable level. In recent years, diverse bacterial communities were assembled to analyze the role of microorganisms in infectious, inflammatory, and metabolic diseases. In this review, we outline their application and features. Furthermore, we discuss the differences between human-derived and model-specific communities. Lastly, we highlight the necessity of generating novel synthetic communities to unravel the microbial role associated with specific health outcomes and disease phenotypes. This understanding is essential for the development of novel non-invasive targeted therapeutic strategies to control and modulate intestinal microbiota in health and disease.

## 1. Introduction

**Figure 1:**
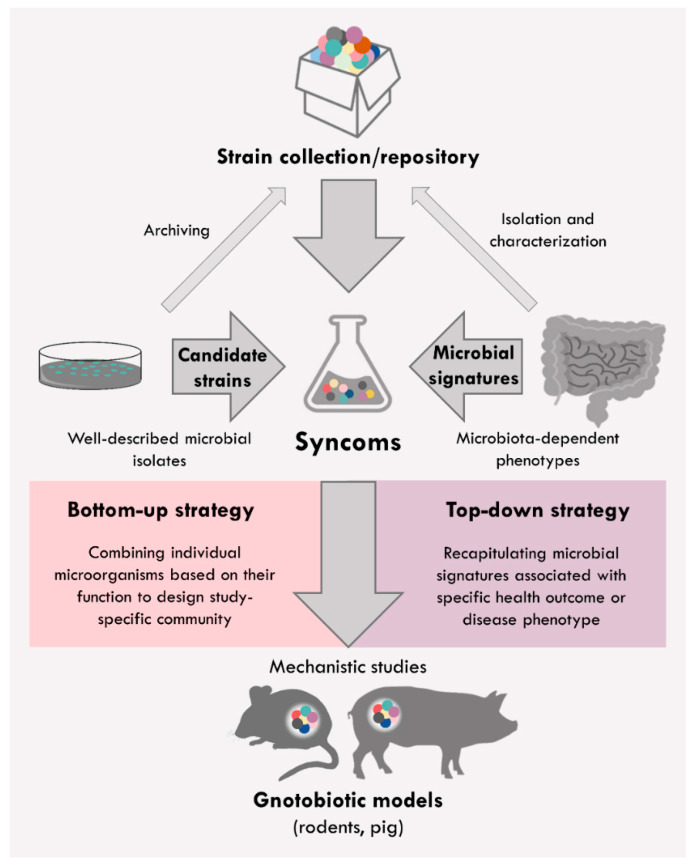
Methodological strategies to assemble syncoms. In a “bottom–up” approach, individual well-described microbial candidates based on their function are combined to assemble study-specific syncoms. In a “top–down” approach, syncoms are assembled to recapitulate observed microbial signatures associated with specific health outcomes and disease phenotypes. The prerequisite for the assemblage of novel communities is the isolation, characterization, and archiving of microbial strains in public repositories. Generated syncoms can be introduced in germ-free animal models such as germ-free rodents and pigs to perform mechanistic or proof-of-concept studies.

The intestine is inhabited by a complex community of microbes that are engaged in a symbiotic/mutualistic interaction with the host. This community participates in many relevant physiological processes for the host such as the maturation of the mucosal immune system and the microbial breakdown of essential food components [1]. Contrarily, shifts in bacterial composition have been associated with the development of multiple human disorders including infectious, inflammatory, metabolic, and autoimmune diseases, as well as with the outcome of colorectal cancer [2,3,4,5,6,7]. However, it is still unclear whether these compositional perturbations are a cause or rather a consequence of a disease. Microbiome-wide association studies performed over the last decade provided a catalog of microbial species that could be used as biomarkers to distinguish healthy population from patients with severe diseases [8,9]. Nevertheless, although the presence of particular microbial genus or species was associated with a certain disease state, studies demonstrating their causative role are still mainly lacking. Thus, to translate microbial correlation into causation, we need to generate models in which functional and mechanistic studies can be addressed. One valuable tool to assess the causal role of microbes in host health and disease is the usage of gnotobiotic animal models [10,11,12]. The word “gnotobiotic” is derived from the Greek “gnotos” (meaning well-known) and “bios” (meaning life). It describes organisms with a fully known microbial status including germ-free animals, which are devoid of all living microorganisms, and animals associated with known microbial species. Although studies in germ-free models are necessary to confirm microbiota-induced phenotype, studies in models with defined microbial composition are particularly appealing, as here, beneficial or detrimental effects of individual microbes or microbial communities can be assessed. Thus, gnotobiotic models represent a unique tool to reduce the complexity of intestinal microbiome sufficiently to perform mechanistic studies. Furthermore, models with this type of microbial standardization can reduce experimental variability and increase experimental reproducibility. The colonization of germ-free animals with minimal microbiomes provides an attractive approach to assess the causality of disease-associated microbial alterations. Hence, it is essential to extend the use and assemble novel synthetic communities (“syncoms”). Syncoms can be employed as both “top–down” and “bottom–up” methodological strategies in microbiome research (Figure 1). In a “top–down” approach, syncoms can be assembled based on the gut community data acquired through the analysis of omics data (e.g., taxonomic diversity, bacterial abundance, dominating taxa, metabolic activity). With this approach, syncoms can be used to recapitulate microbiome signatures observed in microbiota-dependent phenotypes to assess their functionality and causality. Based on these signatures, health- or disease-associated taxa can be isolated, and a novel simplified community can be assembled. The “bottom–up” strategy relies on combining individual microorganisms that are previously cultured and well characterized (e.g., their taxonomic affiliation, ecological niches, individual metabolic profiles, or interactions). Based on a study-specific hypothesis, individual microbial species with particular features can be combined to assemble study-specific syncoms [13]. However, the prerequisite for the extended usage and assembling of novel syncoms is the isolation and cultivation of host-specific microbial taxa and their availability in public strain collections [14,15,16].

In this review, we provide an overview of existing synthetic gut microbiomes/communities and their application in microbiome research to decipher mechanisms underlying host–microbiota interactions in health and disease. In particular, we focus on in vivo models that integrate a supplementary level of complexity and knowledge, i.e., the host responses, in contrast to a recent review by Mabwi et al. that is focused on ex vivo culturomics of synthetic gut microbiota and mathematical modeling [17].

## 2. Syncoms Representing Simplified Intestinal Microbiome

The first attempt to create a synthetic community was undertaken to standardize hygienic conditions in rodent breeding facilities. Russel W. Schaedler performed this pioneer work and established a defined mixture of six cultivable bacterial strains isolated from Nelson Collins Swiss mice [18]. The selection of the Schaedler flora members was based on their cultivability in the laboratory setting [19]. This first syncom included two strains of *Lactobacilli*, one strain of anaerobic *Streptococcus* Group N, two strains of *Bacteroides*, and a coliform strain [18]. The disadvantage of this minimal community was the presence of facultative anaerobes, which outgrew microbial contaminants [20]. Thus, this minimal consortium was modified in 1978 by Roger P. Orcutt to enable better hygiene monitoring. In this mixture, facultative anaerobes were replaced with obligate anaerobes representing the major microbiota constituents of the mouse gut [21]. This refined microbiota was called altered Schaedler flora (ASF) and included eight anaerobic bacterial strains *Clostridium* sp. ASF356, *Lactobacillus acidophilus* ASF360, *Lactobacillus murinus* ASF361, *Mucispirillum schaedleri* ASF457, *Eubacterium plexicaudatum* ASF492, *Pseudoflavonifractor* sp. ASF500, *Schaedlerella arabinosiphila* ASF502, and *Parabacteroides goldsteinii* ASF519 [22]. By inoculating germ-free mice with these bacterial species, a few of the anatomical abnormalities caused by housing under germ-free conditions can be normalized such as cecum size and fecal consistency [18]. However, compared to a complex endogenous microbiota, the ASF syncom is not able to exert colonization resistance and does not recapitulate all major functions of the enteric microbiota. Several studies have shown that introduced microbes including viruses and bacteria can successfully colonize the gut of ASF-associated mice [23,24,25]. This implies that these mice are also highly susceptible to contamination by environmental bacteria and fungi [26], especially if aseptic condition in animal handling by scientists and animal caretakers is not strictly kept. Even though the ASF is widely used in biomedical research, individual bacterial species are not available in public strain collections, complicating the study-based modification of the consortium. In 2020, a novel defined syncom was created by combining some of the ASF members with representative bacteria isolated from specific-opportunistic-pathogen-free C57BL/6J mice including four species obtained from the public repository “German Collection of Microorganisms and Cell Cultures” [27]. This minimal community was named GM15 and includes 15 gut-derived bacterial species (*Lactobacillus johnsonii* MD006, *Lactobacillus murinus* MD040, *Limosilactobacillus reuteri* MD207, *Parabacteroides goldsteinii* MD072, *Bacteroides acidifaciens* MD185, three *Lachnospiraceae* sp. strains MD308, MD335, and MD329, *Bacteroides caecimuris* MD237, *Schaedlerella arabinosiphila* (ASF502) MD300, *Clostridium* sp. (ASF356) MD294, *Enterocloster clostridioformis* YL32, *Clostridium cocleatum* I50, *Escherichia coli* Mt1B1*,* and *Anaerotruncus colihominis* JM4-15) [27]. The GM15 community is described as an innovative gnotobiotic model to standardize laboratory rodent husbandry that increases the reproducibility and robustness of preclinical studies. The rationale behind GM15 assembly was the stable colonization of germ-free mice, comparability with indigenous mouse gut microbiota at the functional level, and simplified monitoring. Oligo-Mouse-Microbiota 12 (OMM^12^) also termed stable defined moderately diverse microbiota (sDMDMm2) is another example of the mouse-derived minimal bacterial microbiome [28,29]. This synthetic bacterial community was developed to decipher the contribution of individual strains to host–microbe and microbe–microbe interactions. OMM^12^ shows typical characteristics of a complex microbiota, as it provides partial colonization resistance against enteric pathogens [29]. It comprises twelve bacterial isolates assigned to five major phyla (*Bacteroidetes*, *Firmicutes*, *Verrucomicrobia*, *Proteobacteria*, and *Actinobacteria*). The selection of bacterial strains was guided by phylogenetic diversity and public availability. All twelve OMM^12^ members (*Akkermansia muciniphila* YL44, *Bacteroides caecimuris* I48, *Muribaculum intestinale* YL27, *Turicimonas muris* YL45, *Bifidobacterium longum subsp. animalis* YL2, *Enterococcus faecalis* KB1, *Acutalibacter muris* KB18, *Enterocloster clostridioformis* YL32, *Blautia coccoides* YL58, *Flavonifractor plautii* YL31, *Limosilactobacillus reuteri* I49, and *Clostridium innocuum* I46) are included in public strain collection of “German Collection of Microorganisms and Cell Cultures”. Furthermore, OMM^12^ characterizes the long-term stability and high reproducibility accomplished in different animal laboratories [29,30]. In a recent study by Streidl et al., this syncom was used for studies addressing host–microbiome metabolic cross-talk. OMM^12^ was modulated with *Extibacter muris*, which is a gut bacterium that metabolizes cholic acid, a form of primary bile acid, into secondary bile acid deoxycholic acid. Secondary bile acids, deoxycholic and lithocholic acid, as well as their taurine conjugates were detected solely in mice co-colonized with *E. muris*. Moreover, this model was able to show that the production of secondary bile acids influenced proteomes in the liver [31]. The production of microbial metabolites such as secondary bile acids can affect inflammatory and metabolic processes. Due to the added microbial function, this extended model consortium could be used to investigate the pathophysiological impact of secondary bile acids on the host. Altogether, OMM^12^ represents an attractive model consortium for targeted mechanistic studies, as it allows flexible experimental design. An overview of the available rodent-derived syncoms and their application in microbiome research is given in Table 1.

Creating human-like conditions in the animal intestine by colonizing germ-free organisms with bacteria isolated from complex human stool samples supports the targeted investigation of important biochemical processes and host–microbe interactions in the digestive tract of people. Humanized animal models mimic more closely the interactions in human intestine and can increase our understanding of microbiota-associated phenotypes of human disorders [39]. Thus, syncoms based on human-derived strains have also been developed (Table 2). The simplified human intestinal microbiota (SIHUMI) consortium is composed of seven bacterial species belonging to the phyla *Firmicutes* (*Anaerostipes caccae* L1-92, *Blautia producta* 2396, *Clostridium ramosum* 113-I, *Lactobacillus plantarum* Lp 39), *Bacteroidetes* (*Bacteroides thetaiotaomicron* E50), *Actinobacteria* (*Bifidobacterium longum* NCC2705), and *Proteobacteria* (*Escherichia coli* K-12 MG1655) [40]. These bacterial species were selected based on their high abundance in the human gut, fermentative abilities, and the ability to form a stable community in the rodent intestine [40]. Colonization with SIHUMI resulted in increased fecal concentration of short-chain fatty acids (SCFA). As the fecal butyrate concentration was low, the butyrate-producing bacterium *Clostridium butyricum* Rowett was added to the SIHUMI consortium to form the extended SIHUMIx microbiota. SIHUMIx syncom recapitulated metabolic features of conventional animals such as SCFA production, as well as mucus, ß-aspartylglycine, and bilirubin degradation [40]. The simplified intestinal microbiota consortium (SIM) is another human-derived syncom composed of ten microbial isolates (*Eubacterium hallii* L2-7, *Eubacterium rectale* A1-86, *Bifidobacterium adolescentis* L2-32, *Collinsella aerofaciens* VPI 1003, *Desulfovibrio piger* VPI 11112, *Roseburia inulinivorans* A2-194, *Ruminococcus bromii* L2-63, *Bacteroides* *thetaiotaomicron* VPI 5482, *Prevotella copri* DSM18205, and *Akkermansia muciniphila* Muc) [41]. This syncom was generated to clarify how bacteria–diet interactions contribute to metabolic disorders. The selection criteria for SIM consortium assembly were dominated abundance in the human gut, a fully sequenced genome available in the public database, and well-characterized metabolic features including complementing properties to metabolize dietary components. Thus, SIM community recapitulates many functions of the complex human microbiota and may facilitate our understanding of how the microbes interact with macronutrients to affect host metabolism in a diet-specific manner [41]. In a similar line, Rezzonico et al. established a humanized gnotobiotic mouse model by choosing ten dominating and easy-to-grow bacteria from stool samples of adult humans (*Bacteroides thetaiotaomicron* E50, *Bacteroides vulgatus* DSM1447, *Bifidobacterium longum* NCC2705, *Blautia hansenii* VPI C7-24, *Clostridium scindens* 19, *Collinsella aerofaciens* DSM3979, *Escherichia coli* HS, *Eubacterium ventriosum* VPI 1013B, *Faecalibacterium prausnitzii* A2-165*,* and *Lactobacillus rhamnosus* NCC4007). The advantage for the use of this consortium in studies is the availability of the microbial members in public bacterial collections and the completely sequenced genome. Metabolic characterization of this syncom showed that the microbial composition affects hosts metabolic profiles, which may play a substantial role in physiological processes and disease development in humans [42]. Other synthetic human bacterial communities to be mentioned here have been developed to investigate inter-bacterial communication and interactions. Welch et al. used a defined 15-member model consortium comprised of phylogenetically diverse and sequenced human gut bacterial strains to explore the spatial organization of the gut microbiota [43]. Imaging analyses of this syncom revealed that luminal and mucosal proximal colon compartments are not sharply stratified but should rather be seen as an incompletely mixed bioreactor [44]. Denou et al. investigated the interactions between three human commensals: *Escherichia coli* K-12, *Lactobacillus johnsonii* NCC533, and *Bifidobacterium longum* NCC2705 [45]. This syncom was used as a model community to study cooperation and competition interactions in the mammalian gut. The introduction of additional microbes such as *E. coli* Nissle strain in this model system allowed studying how different gut species coexist or are eliminated due to the provided colonization resistance. Furthermore, by studying this simple interacting bacterial population, factors governing the microbial ecology and dynamic within the gastrointestinal tract can be determined. Similar human-derived syncom comprised of three prominent *Bacteroides* species (*Bacteroides ovatus* ATCC 8483, *B. vulgatus* ATCC 8482, and *B. thetaiotaomicron* VPI 5482) was assembled to analyze cooperation interactions within the *Bacteroidales*, which is the dominant Gram-negative bacterial taxa of the human intestine [46]. This study provided evidence of a distinct form of cooperativity within the *Bacteroidales* taxa that represent an example of naturally evolved cooperative interactions between the microbes [46]. The ability of microbes to utilize the waste products of another species is well-known example of cooperation interactions [47]. However, in this study, a cross-feeding enzyme system in the gut symbiont *B. ovatus* was discovered, which digests inulin at a benefit to another species, as these enzymes are unnecessary for its use of inulin. By the extracellular digestion of inulin, *B. ovatus* feeds other gut species, which in return provide benefits to *B. ovatus*. These data support the hypothesis that these strong eco-evolutionary interactions within the microbiota are essential for the ecological stability and functioning of intestinal communities [46].

When selecting synthetic consortia to conduct studies, it is important to consider not only whether to use human-derived or host-specific community but also which life stage is addressed in this study. The majority of available minimal microbial consortia recapitulate the microbial composition or microbial signatures found in the adult fecal microbiome. However, the microbial composition of the gut microbiota differs greatly between different age stages due to alimentary and environmental factors [63,64,65]. Therefore, it is important to assemble age-specific consortia as well as to distinguish between the infant, adult, and elderly microbiome. A few years ago, Luk et al. generated a simplified model community of the human infant gut microbiota comprised by four *Bifidobacterium* species with high abundance in healthy infants but low abundance in adults [55]. This infant-specific syncom includes *Bifidobacterium longum subsp. infantis* S12, *Bifidobacterium breve* S50 (Variant a), *Bifidobacterium bifidum* Ti, and *Bifidobacterium dentium* B757. Using this syncom, the authors investigated the effects of infant-type *Bifidobacteria* on brain function including recognition memory, as early-life gut colonization was shown to be involved in the development of the central nervous system via the microbiota–gut–brain axis [66]. The study results demonstrated that neonatal colonization with the human infant microbiota containing *Bifidobacteria* exerted similar beneficial neuromodulatory effects in gnotobiotic mice as colonization with complex microbiota. Moreover, *Bifidobacteria* particularly improved recognition memory [55]. These findings of bacteria-derived beneficial effects on the brain may contribute to the development of novel microbiota-based therapeutic interventions for human neurodevelopmental disorders.

Altogether, synthetic microbiomes represent modular systems that allow various combinations of intestinal isolates based on their function to mimic physiological conditions of interest.

## 3. Utilizing Syncoms to Model Gut Disease Phenotypes

Apart from the synthetic communities mimicking indigenous gut microbiota, disease-specific consortia have been assembled for application in germ-free animals to identify and define the causal role of bacteria in the pathogenesis of human disorders. In the next paragraphs, we give an overview of existing human- and rodent-derived syncoms utilized in mechanistic studies to untangle disease-associated host–microbe interactions (Table 1 and Table 2). Moreover, here, we only focus on the development of enteropathies categorized in infectious, inflammatory, and metabolic diseases as well as colorectal cancer.

### 3.1. Infectious Diseases

The intestinal microbiota prevents the overgrowth of potentially pathogenic microorganisms and thus protects intestinal tissues from microbial invasion [67,68]. However, diverse environmental conditions, such as antibiotic application or contaminated food, can damage the stability and functionality of the intestinal barrier. The resulting enteric impairment can lead to the typical symptoms of infectious colitis such as nausea, vomiting, and diarrhea. Even though infection rates in the Western world have reduced dramatically over the past century due to advances in hygiene, antibiotics, and vaccination, infectious diseases of the gut induced by bacteria, viruses, or even parasites are still among the most frequent causes of medical treatment worldwide [69]. A typical secondary manifestation after antibiotic treatment is, e.g., the overgrowth of *Clostridioides difficile* (former *Clostridium difficile*) in the intestine, which can lead to infectious colitis due to the impaired intestinal defenses [70,71]. Although OMM^12^ shows typical characteristics of the normal complex microbiome by providing colonization resistance against some enteric pathogens such as *Salmonella enterica* serovar Typhimurium, it does not exhibit resistance to *C. difficile* infection (CDI) [36]. Metabolic analyses of OMM^12^ members confirmed the deficiency for the 7α-dehydroxylation, bacterial transformation producing secondary bile acids, deoxycholic and lithocholic acid [36]. These microbial metabolites are associated with protection against pathogen colonization such as *C. difficile* [72]. Modulation of OMM^12^ with the 7α-dehydroxylating commensal *Clostridium scindens* strain ATCC35704 resulted in physiological bile acid composition in the large intestine and protected against CDI. Interestingly, secondary bile acids are absent in mice monocolonized with *C. scindens*, indicating that this *Clostridium* species requires the presence of other intestinal microbes for certain metabolic processes [73]. As *C. scindens* can only metabolize unconjugated bile acids, the deconjugation of bile acids was carried out by the OMM^12^ community, which in turn resulted in transient protection against CDI [36]. This study describes how already established syncoms can be modified to generate communities specifically tailored for the study demand. Furthermore, metabolic cross-talk between commensals and the host can be explored in more detail in models associated with syncoms. Crost et al. investigated defense mechanisms of the intestinal microbiota against pathogenic *Clostridium* species. In this study, the authors established a syncom containing bacteria that are involved in the colonization resistance against the opportunistic pathogen *Clostridium perfringens,* which causes food-borne disease in humans, to identify metabolites mediating this protective effect [56]. This simplified syncom included four cultivable bacteria *Ruminococcus gnavus* E1, *Bacteroides thetaiotaomicron* LEMF4, *Clostridium hathewayi* LEMC7, and *Clostridium orbiscindens* LEMH9, and it was able to stably colonize the digestive tract of rodents. To identify metabolites mediating colonization resistance, different combinations of these bacteria were challenged with *C. perfringens* in vivo. A diffusible trypsin-dependent bactericidal substance produced by the *R. gnavus* E1 strain was identified as a key metabolite for *C. perfringens* elimination. However, this protective effect mediated by *R. gnavus* E1 was dependent on the presence or absence of other bacteria in the consortium [56]. Another mechanism of protection from infectious enteric diseases is to strengthen the host’s intestinal defense. The Microbial Ecosystem Therapeutic (MET-1) consortium was developed by isolating 33 bacteria from healthy human stool. This consortium was initially used to cure patients with recurrent CDI. Martz et al. hypothesized that MET-1 might also protect against other systemic infectious agents, such as *S. enterica* serovar Typhimurium. In this study, the authors found that MET-1 does not inhibit the growth of *S. enterica* serovar Typhimurium in the intestine. However, the application of MET-1 strengthened the host intestinal barrier function by increasing tight junction gene expression, attenuating the infiltration of immune cells in the intestinal mucosa, and reducing the local expression level of pro-inflammatory cytokines. As a result, the intestine was protected from severe tissue damage, and systemic spread of the bacteria was prevented [54]. Thus, MET-1 syncom could be used as novel strategy of microbiota-based therapy to protect against the severe disease outcome after *S. enterica* serovar Typhimurium infection. In one other study using gnotobiotic mice associated with the OMM^12^ consortium, bacterium *M. schaedleri* was found to protect a genetically predisposed host against *Salmonella*-induced colitis. The protective effect mediated by *M. schaedleri* involved the inhibition of *Salmonella* virulence factor expression and competition for anaerobic respiration substrates in the gut [38]. However, certain bacterial composition of intestinal microbiota may not always exert a beneficial effect on the host but may also be associated with the development of enteric disorders or their exacerbation. By using a well-defined gnotobiotic mouse model colonized with SHUMIx, Ganesh et al. investigated whether an additional enteric commensal *A. muciniphila* influences the inflammation induced by *S. enterica* serovar Typhimurium [40,51]. *A. muciniphila* belongs to the *Verrucomicrobia* phylum and has mucolytic property through the enzymatic degradation of mucin [74]. Thus, it is hypothesized that *A. municiphila* can play a role in the development of intestinal inflammation. Indeed, the co-colonization of *A. muciniphila* in *S. enterica* serovar Typhimurium-infected SIHUMIx mice caused exacerbated colon histopathology and increased levels of pro-inflammatory cytokines. Additionally, vast alterations of the SIHUMIx bacterial composition were caused by the intestinal presence of *A. muciniphila* and *S. enterica* serovar Typhimurium. In the presence of *A. muciniphila*, *S. enterica* serovar Typhimurium was able to suppress the dominating bacterial taxa *Bacteroides thetaiotaomicron* [51]. Due to excessive mucin degradation by *A. municiphila*, luminal antigens can gain access to the intestinal mucosa and stimulate the host immune system and the production of antimicrobial peptides that can in turn cause changes in the microbiota composition. On the other hand, due to mucus layer degradation by *A. muciniphila, S. enterica* serovar Typhimurium can more easily reach the underlying tissue and induce inflammatory lesions. However, the microbial capacity to exacerbate acute intestinal inflammation is not only a species-specific feature but is also influenced by the bacterial strain. The symbiont *Escherichia coli* is commonly found in the digestive tract of mammals worldwide. Although this bacterium is an obligate component of healthy human microbiota, it can also cause enteric and extraintestinal disease, depending on the strain of *E. coli*. Thus, Kittana et al. analyzed the effects of different *E. coli* strains isolated from healthy conventional mice in a chemically triggered gnotobiotic mouse model of inflammatory bowel disease (IBD). While some *E. coli* strains (isolates ST150 and ST468) exerted a neutral effect on induced inflammation in ASF colonized mice, other strains (isolates ST129 and ST375) exacerbated inflammatory lesions in the gut [33]. This observation indicated that not only the species but also the strain should be considered in host–microbiota studies, since different strains can interact differently with the host resulting in neutral, beneficial, or detrimental effect.

### 3.2. Inflammatory Diseases

In many experimental models of intestinal inflammation, animals are free of disease-specific signs under germ-free conditions indicating that the gut microbiota is crucial for disease pathogenesis [75,76,77,78]. To determine whether single or even groups of microbes affect the onset and severity of the disease, functional studies using defined intestinal consortia are required. Using the SIHUMIx community, Ring et al. investigated whether *A. muciniphila* strain ATCC BAA-835 promotes the development of intestinal inflammation [50]. There are contradictory studies reporting both beneficial and detrimental effects of this bacterium in the context of intestinal inflammation [51,79,80]. Furthermore, the intestinal abundance of *A. muciniphila* is mainly associated with beneficial effects in terms of metabolic disease outcome [81,82,83]. The colonization of germ-free interleukin 10 (IL10)-deficient mice, a model for experimental IBD, with SIHUMIx syncom caused histopathological changes in the intestine. Amendment of the SIHUMIx consortium with *A. muciniphila* did not exacerbate colitis nor influence the abundance of SIHUMIx members [50]. This study confirmed that the *A. muciniphila* ATCC BAA-835 strain does not promote chronic intestinal inflammation in gnotobiotic mice genetically predisposed to colitis. Eun et al. developed another SIHUMI microbiota to study IBD-related microbiota–host interactions. The bacteria were selected based on their altered abundance in patients with IBD and healthy individuals, ability to affect experimental colitis, human origin, and ability to stably colonize rodents [52]. The community is composed of seven bacterial taxa including *Enterococcus faecalis* OG1RF, *Ruminococcus gnavus* ATCC 29149, *Faecalibacterium prausnitzii* A2-165, *Lactobacillus plantarum* WCFS1, *Bacteroides vulgatus* ATCC 8482, *Escherichia coli* LF82, and *Bifidobacterium longum subsp. longum* ATCC 15707. When this consortium was introduced into germ-free experimental models of IBD, it promoted colitis development [52]. The colitogenic contribution of *E. faecalis* within the SIHUMI consortium was characterized by comparing SIHUMI-associated mice lacking this bacterium with animals carrying the complete syncom [53]. Surprisingly, the deletion of *E. faecalis* resulted in more severe colitis characterized by increased histopathological tissue lesions and higher expression levels of pro-inflammatory cytokines, suggesting that this opportunistic pathogen provides a beneficial effect within this IBD-related consortium. Moreover, this observation demonstrated that interactions with co-colonizing bacteria may reprogram the pro-inflammatory activity of *E. faecalis* [53]. Viral infections such as norovirus infection were also associated with exacerbated colitis symptoms in experimental models of colitis as well as IBD patients [34,84,85,86]. However, studies using gnotobiotic models or depleted microbiota demonstrated that the colitogenic stimulus of norovirus depends on the presence of enteric microbiota [34,84]. Norovirus infection exacerbated intestinal inflammation in both ASF syncom and complex microbiota colonized IL10-deficient mice. In contrast, germ-free IL10-deficient mice did not develop colitis after norovirus infection [34]. A subsequent study investigated the contribution of bacterial composition to viral-triggered intestinal inflammation by comparing two different syncoms, ASF and OMM^12^ [23]. In this study, norovirus infection exacerbated colitis only in IL10-deficient mice carrying ASF but not those colonized with OMM^12^. Additionally, the modulation of these syncoms with immunomodulatory segmented filamentous bacteria (SFB) abolished intestinal inflammation in mice associated with ASF despite norovirus infection. The SFB-mediated protective effect was associated with enhanced intestinal barrier defenses [23]. Altogether, this showed that the composition of intestinal microbiota determines the outcome of disease.

In terms of host-mediated factors, the impaired intestinal epithelial barrier function in IBD patients allows the entry of luminal bacteria into the gut tissue, inducing an inflammatory immune response [87,88]. To address the mechanisms causing chronic colon inflammation in response to epithelial barrier defects, Eftychi et al. used gnotobiotic mice with intestinal epithelial cell-specific deficiency of nuclear factor-κB essential modulator (NEMO). Colitis development in this model is microbiota-driven. In this study, the non-pathogenic commensal syncom ASF alone was sufficient to trigger chronic inflammation by promoting the release of pro-inflammatory cytokines IL-12, IL-23, and IFN-γ. In the early stages of the disease, colitis was driven by IL-12, whereas the inflammation in later stages was driven by IL-23. These results suggest that similar mechanisms might contribute to IBD pathogenesis, especially in patients with impaired intestinal barrier [32]. Due to its crucial role in the development of inflammation, modulation of the gut microbiota holds great promise for the treatment of patients with chronic inflammatory disorders. As an example of a therapeutic syncom, Atarashi et al. assembled a defined, complex bacterial mixture of *Clostridium* strains, particularly clusters IV and XIVa isolated from human stool [57]. These bacteria were selected based on their high potency in enhancing regulatory T cell immune response, driving the resistance to IBD and allergies. The underlying molecular mechanism was attributed to the production of bacterial metabolites such as SCFA, which induce regulatory T cells in the colonic mucosa [89]. The characterization of these metabolites, including their mechanistic interaction with the host, may allow targeted therapeutic manipulation of intestinal dysbiosis.

### 3.3. Metabolic Disorders

The gastrointestinal tract of healthy individuals serves as a biotope and nutritional source for varieties of microbial species. In this symbiotic relationship, the host benefits from hundreds of bacterial genes not present in the human genome that enable the extraction of essential nutrients from food that would otherwise not be used by the body [1]. The bacterial production of metabolites such as beneficial SCFA (butyrate, acetate, and propionate) is a main source of energy for the host colonocytes, but these are also required for the maintenance of the intestinal and immune homeostasis [90]. As microbial-derived metabolites have an immense modulating impact on intestinal physiology and host metabolism, dysbiosis-associated changes in microbiota composition can lead to the development of metabolic diseases [5]. In this context, the composition of the human microbiome is shaped by environmental factors such as antibiotic treatments, stress, and unhealthy diet, which can cause dysbiosis, leading to a breakdown of intestinal homeostasis [91,92,93,94]. Thus, due to increasing incidence of metabolic diseases such as type 2 diabetes and obesity, research efforts focus on deciphering the influence of diet in shaping the microbiome. Each bacterium has its metabolic profile and, depending on the availability of macromolecules derived from diet, the composition and diversity of the microbiota change [95,96,97]. However, so far, only an association between the prevalence of certain diseases and specific eating habits has been established. For example, a Western diet (obesity-associated diet) rich in carbohydrates and fats causes an adaptive shift in the abundance of bacterial communities by favoring those having increased capacity to harvest energy from the diet [98]. Thus, the characterization and modulation of microbial metabolic activity could help to create novel therapies for treating metabolic diseases. Hence, the use of gnotobiotic animal models in combination with defined nutritional formulas is essential to explore diet-derived changes of the microbiota and their influence on the energy balance. This approach allows hypothesis-driven, simplified, and controlled studies under highly standardized microbial conditions. The SIHUMIx consortium represents a valuable and frequently used gnotobiotic model to explore metabolic dysfunction, particularly with regard to obesity [40]. Using this syncom, it was demonstrated that the SIHUMIx member *C. ramosum* promotes high-fat diet-induced obesity [48]. The underlying mechanism of the bacteria-related obesogenic potential was attributed to the upregulation of glucose and fat transporters in the small intestine, resulting in increased diet digestibility and body fat deposition. Furthermore, Woting et al. also investigated whether *B. longum* within the SIHUMIx consortium was able to mediate the diet-induced improvement of obesity symptoms. Previous studies reported that the oligofructose ameliorates obesity by promoting the growth of intestinal *Bifidobacteria* [99,100]. Woting et al. showed that the oligofructose supplementation reduced obesity by decreasing body fat accumulation and improving glucose tolerance, but this beneficial effect was independent of the presence of *B. longum* [101]. Using the same community, Weitkunat et al. investigated the effects of dietary fibers on lipid metabolism [49]. An excess production of SCFA derived from the microbial fermentation of indigestible fibers was reported to promote obesity [102,103]. Thus, gnotobiotic mice associated with SIHUMIx were fed a high-fat diet supplemented with either fermentable (inulin) or non-fermentable (cellulose) fibers [49]. As inulin serves as a substrate for enteric bacteria, feeding the inulin-supplemented diet affected the intestinal SIHUMIx consortium. All SIHUMIx members except *E. coli* and *L. plantarum* increased in numbers, leading to increased SCFA production with a reduced cecal acetate/propionate ratio compared to cellulose-fed mice. Interestingly, additional energy extraction from the inulin diet did not increase fat deposition and body weight but led to an increased bacterial proliferation. Furthermore, inulin feeding positively influenced lipid metabolism by decreasing the hepatic expression of genes involved in lipogenesis and fatty acid elongation/desaturation, changing plasma and liver phospholipid composition, and increasing omega-3:omega-6 fatty acid ratio. Altogether, these data suggest beneficial effects of inulin on preventing obesity [49]. Although dietary cellulose had no effect on the outcome of obesity, another study demonstrated its impact on components of the intestinal immune system resulting in the inhibition of colitis induction [37]. By feeding conventional mice fiber-free diets, the intestinal abundance of *Alistipes* genus was strongly reduced. To investigate the causal relationship of this bacterium to cellulose-mediated protection, Oligo-MM^12^ syncom was refined by adding *Alistipes finegoldii*, which is a bacterium that can metabolize cellulose into glucose. In this system, dietary cellulose modified the gene expression of the intestinal epithelium in favor of anti-inflammatory immunity, ensuring intestinal homeostasis. Thus, the co-colonization of Oligo-MM^12^ syncom with *A. finegoldii* decreased the sensitivity to chemically induced colitis development. Analyses of colonic tissue revealed minimized inflammatory lesions that were the result of the strengthened intestinal barrier function through the increased expression of regulatory cytokines and epithelium-enhancing proteins [37]. Altogether, this study supports the reported health-promoting effects of dietary cellulose by providing causal mechanisms in a gnotobiotic animal model. McNulty et al. analyzed the impact of dietary supplements in terms of commercially available probiotics on the indigenous gut microbiome [43]. For this purpose, a synthetic minimal microbiome containing 15 sequenced gut symbionts belonging to three principal bacterial phyla of human microbiota (six *Bacteroides* species, two *Clostridium* species, *Collinsella aerofaciens*, *Dorea longicatena*, *Eubacterium rectale*, *Faecalibacterium prausnitzii*, *Parabacteroides distasonis*, and two *Ruminococcus* species) was assembled. Microbial species were selected based on their representative abundance in the human gut and their functional similarity to microbial features observed in the healthy adult fecal microbiome. Gnotobiotic mice associated with the 15-member syncom were treated with probiotic fermented milk product (FMP) containing *Bifidobacterium animalis subsp. lactis*, two strains of *Lactobacillus delbrueckii subsp. bulgaricus*, *Lactococcus lactis subsp. cremoris*, and *Streptococcus thermophilus*. Metatranscriptomic and metabolic analyses were performed to characterize the impact of the probiotic consortium on the established model microbiome. Members of FMP successfully colonized the murine intestine, causing only minor shifts in the indigenous community. Furthermore, the introduced FMP bacteria enriched the model syncom by providing microbial enzymes involved in diverse metabolic processes such as the fermentation of specific carbohydrates. These experiments showed that synthetic microbiomes can be used as a translational pipeline for characterizing the effects of FMPs on the human microbiome [43]. In a further study by the same research group, the model human consortium comprising now twelve sequenced gut bacterial species was used to analyze the effect of diet on endogenous microbiota. Gnotobiotic mice colonized with artificial human-derived community were fed by oscillating diets of contrasting composition (low-fat/high-plant polysaccharide and high-fat/high-sugar diet) that induced rapid, reproducible, and reversible changes in the microbial community structure [58]. Thus, this bacterial consortium can also serve as a model human gut community providing similar metabolic functions to describe the interactions of indigenous intestinal microbes with environmental factors such as diet. Depending on the research hypothesis and investigated dietary formula, the model human consortium was accordingly modified in its microbial composition to dissect the metabolic niche and contribution of each community member to the host metabolism [59,60]. Narushima et al. designed a six-member syncom, B4PC2, by combining human fecal isolates capable of metabolizing bile acids (*Bacteroides uniformis*, *Bacteroides vulgatus*, *Clostridium hylemonae* TN-271, *Clostridium hiranonis* TO-931, *Parabacteroides distasonis*, and *Blautia producta*). Experiments employing this human-derived syncom demonstrated that human gut microbes can metabolize conjugated primary bile acids into secondary bile acids in vivo [61]. By revisiting this model using omic technologies, the transcriptional profiles of each bacterium within this consortium were defined, revealing the expression of bacterial genes involved in bile acid metabolism [62]. Bile acids not only affect the structure and function of the gut microbial community but also play an important role in host health and disease such as gallstone disease, IBD, and CDI [93,104,105,106]. As the ingested food/diet affect the gut microbiota and the host bile acid signature, it is important to decipher interactions between diet and intestinal microbiota to create new dietary guidelines for individuals of various life stages and health status.

### 3.4. Colorectal Cancer

Several studies using humanized cancer-related animal models after fecal microbiota transfer showed that tumor incidence is strongly associated with the bacterial composition of the human inoculum and independent of the donor health status [107]. These findings indicated that the initial community structure of the gut microbiome of the recipient mice might trigger colorectal cancer. The complex fecal microbiota transfer in germ-free mice is still predominantly used as a model to assess microbiota contribution to the development of colorectal cancer [108,109,110,111,112]. However, to determine the specific contribution of individual bacteria to colorectal cancer development, it is important to create cancer-related syncoms providing controlled and standardized research conditions. In this line, Donohoe et al. used a gnotobiotic mouse model associated with four commensal members of the ASF consortium, which were fed with a high-fiber diet to demonstrate that fibers do have a potent tumor-suppressive effect [35]. By amending the four-ASF syncom with the butyrate-producing bacterium, *Butyrivibrio fibrisolvens*, the gnotobiotic mice developed fewer and less-advanced colonic tumors. However, fibers suppressed colorectal cancer development in a microbiota- and butyrate-dependent manner [35]. Even though the number of studies using syncoms in the current colorectal microbiome research is still sparse, this model has a great potential to determine the ability of particular microbiota members to modulate colorectal cancer development.

## 4. Differences between Human-Derived and Model-Specific Communities

In the above sections, the use of different syncoms and their modulations in the microbiome research was described. However, the described syncoms not only differ in their bacterial composition and function but also in the origin of the selected bacteria. Thus, in this section, the advantages and limitations of human-derived or model-specific communities will be addressed.

**Figure 2:**
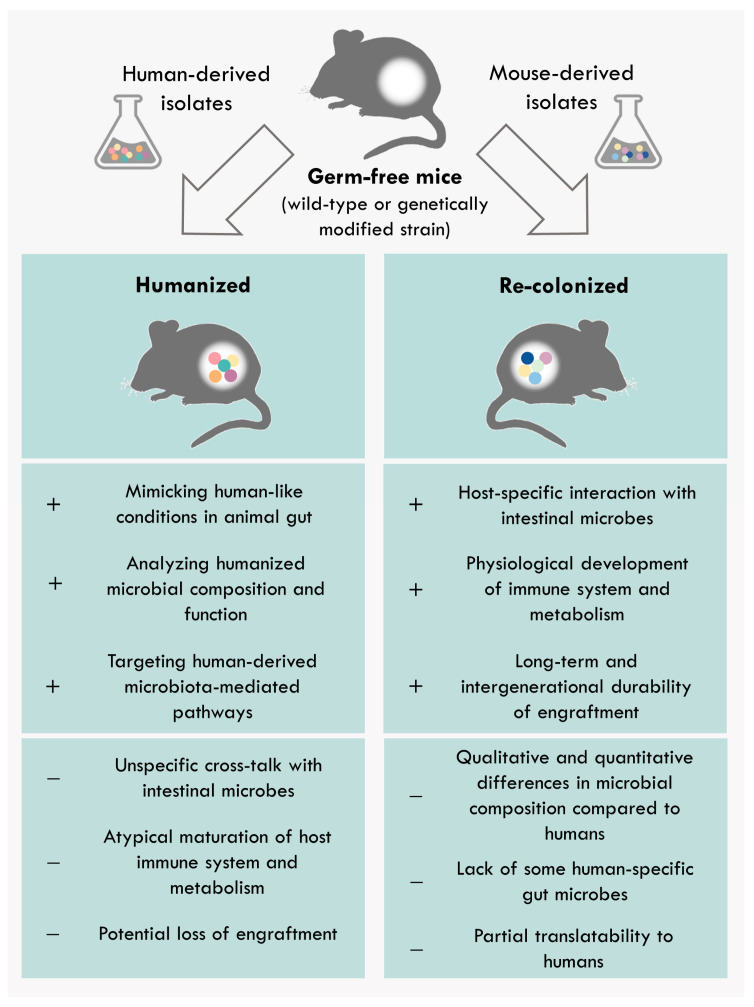
Advantages/limitations of gnotobiotic animals colonized with human-derived or model-derived communities. Predominantly used gnotobiotic animal models are gnotobiotic mice. Gnotobiotic mice are highly controlled in regard to their microbial environment, genetics, and diet. Germ-free mice can be associated with human-derived or mouse-derived communities to perform mechanistic studies to decipher microbial role in the host physiology or pathology under highly standardized conditions.

The use of human-derived bacterial consortia in gnotobiotic animal models allows performing standardized experiments that cannot be performed in humans. Currently, the predominantly used gnotobiotic animal models are gnotobiotic mice (Figure 2). In this context, gnotobiotic models are highly controlled in regard to their microbial environment and diet as well as genetically standardized by using inbred strains. Additionally, numerous genetically modified mouse strains are available, which allow mechanistic studies of particular molecules and pathways. Despite some limitations, the application of gnotobiotic mouse models colonized with human-derived microbes contributes to the deciphering of the functional role of human microbiota. Although the gut microbiota between humans and laboratory mice is qualitatively similar, there are large quantitative differences [113]. Studies showed that the relative proportion between *Firmicutes* and *Bacteriodetes* (F/B ratio) has an impact on human health status, as shifts in this ratio were associated with the development of different disorders, such as obesity (increased F/B ratio) and IBD (decreased F/B ratio) [114,115]. However, the physiological F/B ratio differs between humans and mice. Phylum-level analyses demonstrated a higher F/B ratio in humans than in mice that potentially could interfere with the interpretation of microbial shifts in humanized mice [116]. Furthermore, some microbes are host-specific and cannot colonize other species [113,117,118]. These inter-species variations also influence the microbial metabolism, resulting in dissimilar bacteria-derived metabolite composition [119,120,121]. Thus, the loss of these host-specific microbial products in humanized mice may affect the host physiology [120]. An important issue to mention here is also the complex host-specific cross-talk between individuals and their microbiota, which influences the development and maturation of the intestinal immunology and physiology [122,123]. Hereby, the enteric colonization shapes the mucosal immune system, which in turn forms the intestinal microbiota [89,124]. Thus, the colonization of non-human hosts with human-derived taxa could lead to an atypical maturation of the host immune system [117]. Additionally, the loss of certain bacterial species or shifts in the microbial abundance was observed when the bacterial consortium was transferred into a host with which it has not co-evolved [125]. However, this host-derived effect was not very pronounced in human-microbiota-associated mice [39,117,125]. One way to circumvent these differences is to assess the functionality of the gut microbiome in a host-specific manner. The prerequisite for this approach is the availability of model-specific collections of microbial isolates. To this end, the mouse (miBC) and pig (piBAC) intestinal bacterial collections were recently created [14,16]. These public repositories include all cultivable bacterial strains from the mouse and pig intestine that are publicly available with the aim of supporting colonization experiments by providing a collection of well-described commensal strains. Moreover, the engraftment of human-specific microbes is improved in species that share more similarities with humans such as gnotobiotic rats or pigs [126]. In this line, a study by Wos-Oxley et al. showed that the colonization efficiency of human taxa is higher in gntotobiotic rats than in gnotobiotic mice [118]. Currently, the use of other gnotobiotic species in microbiome research is still limited, which is mainly due to the high maintenance costs, lower reproduction rate, as well as restricted availability of knockout models and reagents such as antibodies. However, their relevance will increase in the future, especially the use of gnotobiotic pigs as a clinically relevant model.

## 5. Conclusions and Future Prospective

The generation of minimal microbiomes and their application in gnotobiotic models allows mechanistic investigations of host–microbe interactions under controlled conditions. However, the development of novel synthetic minimal communities, as well as the refinement of already existing ones to the latest scientific findings, is essential to untangle the underlying mechanisms of host–microbiota interactions and their role in host health and disease. Syncoms represent a valuable compromise between the non-translatability of colonization with single microbial species and the huge complexity of conventional unknown microbiota. The aim of minimal microbiomes utilization is to reduce this complexity to a manageable level, allowing addressing well-defined functional questions and demonstrating the causality of microbiota-associated phenotypes. Thus, the field is streaming to design representative minimal microbiomes that can recapitulate functions of the complex microbiomes. Studies have shown that the intestinal microbiota harbors important keystone taxa, which have a pivotal role in the ecosystem dynamics by dominating inter-microbial interaction networks. In this context, these certain keystone species influence community composition, functionality, and stability, as their absence can lead to intestinal imbalances [127]. In the case of disease-associated syncoms, the microbial community should also best reflect the phenotype, including clinical signs, tissue-specific pathology, and similarities in pathogenesis. Compared to complex fecal microbiota transfer using stool samples with unknown microbiota composition, the application of syncoms is controllable, standardized, and reproducible because of the defined microbial composition based on specific bacterial features, not only the donor’s phenotype. Additionally, the pure bacterial mixture is more stable than the fecal inoculum for the colonization of germ-free animals [30,128]. However, by simplifying intestinal microbial communities, the risk of not recapitulating microbiota signatures and phenotypes increases. To prevent the loss of microbiota-mediated phenotypes, it is essential to design more complex syncoms. In this context, the prerequisite for refining synthetic microbiota is the isolation and characterization of still uncultured microbial species, not only bacteria, but also viruses, fungi, phages, and archaea that are part of the indigenous complex microbiota. Despite the high inter-individual variability, up to 65% of molecular species detected by the sequencing of mouse and human complex gut communities have representative strains in culture. However, numerous taxa representing a source of novel functions are still undiscovered [129,130]. Furthermore, the augmentation of syncoms with non-bacterial species that affect host metabolism, immune response, and physiological processes will improve the functional recapitulation of complex microbial communities. Thus, it is essential to further support initiatives aiming to establish or maintain already existing collections and databases of human and model-specific microbial isolates [14,15,16,131,132]. The availability of well-described intestinal microbial isolates will contribute to the creation of robust experimental models in which generated hypotheses can be tested by systemic manipulations of specific variables. Hence, the combination of gnotobiotic and multi-omic approaches—particularly, transcriptomics, metabolomics, and proteomics—will provide improved insight into the cause–effect relationship between the microbes and the host that is a prerequisite to efficiently translate microbiome research into clinical application. Comprehensive understanding of host–microbe and microbe–microbe interactions is fundamental for the development of novel therapeutics, which will enable a non-invasive targeted modulation of the intestinal microbiota. Future medication could consist of e.g., microbial metabolites or derivatives that effectively abolish intestinal imbalance while reducing the systemic side effects compared to antibiotics.

## Figures and Tables

**Table 1:** Overview of the application of rodent-derived syncoms in microbiome research.

Syncom	Composition	Application/Research Question	Ref.
ASF	*Clostridium* sp. ASF356, *L. acidophilus* ASF360, *L. murinus* ASF361, *M. schaedleri* ASF457, *E. plexicaudatum* ASF492, *Pseudoflavonifractor* sp. ASF500, *S. arabinosiphila* ASF502, *P. goldsteinii* ASF519	Standardization of laboratory rodent husbandry	[21]
		Impact of host-mediated factors on chronic inflammation	[32]
	+*E. coli* strains	Impact of microbiota on intestinal inflammation	[33]
	+Murine norovirus +Segmented filamentous bacteria		[23,34]
	*L. acidophilus* ASF360, *L. murinus* ASF361, *M. schaedleri* ASF457, *P. goldsteinii* ASF519, *B. fibrisolvens*	Diet-related microbial protection against colorectal cancer	[35]
GM15	*L. johnsonii, L. murinus, L. reuteri,* *P. goldsteinii, B. acidifaciens,* 3× *Lachnospiraceae* sp. strains, *B. caecimuris, S. arabinosiphila* ASF502, *Clostridium* sp. ASF356, *E clostridioformis* YL32, *C. cocleatum, E. coli, A. colihominis*	Standardization of laboratory rodent husbandry	[27]
OMM^12^	*A. muciniphila* YL44, *B. caecimuris* I48, *M. intestinale* YL27, *T. muris* YL45, *B. longum subsp. animalis* YL2, *E. faecalis* KB1, *A. muris* KB18, *E. clostridioformis* YL32, *B. coccoides* YL58, *F. plautii* YL31, *L. reuteri* I49, *C. innocuum* I46	Mechanisms of colonization resistance against enteric pathogens	[29]
	+*E. muris*	Host–microbe metabolic cross-talk and bile metabolism	[31]
	+*C. scindens*	Mechanisms of colonization resistance against *C. difficile*	[36]
	+Murine norovirus +Segmented filamentous bacteria	Impact of microbiota on intestinal inflammation	[23]
	+*A. finegoldii*	Host–microbe metabolic cross-talk and diet impact on inflammation	[37]
	+*M. schaedleri*	Impact of microbiota on *Salmonella*-induced intestinal inflammation	[38]

**Table 2:** Overview of the application of human-derived syncoms in microbiome research.

Syncom	Composition	Application/Research Question	Ref.
SIHUMI	*A. caccae, B. producta, C. ramosum,* *L. plantarum, B. Theta*^1^, *B. longum, E. coli* K-12	Host–bacteria interactions	[40]
SIHUMIx	*A. caccae, B. producta, C. ramosum, L. plantarum, B. theta*^1^, *B. longum, E. coli* K-12, *C. butyricum*	Host–microbe metabolic cross-talk and impact of microbiota on intestinal inflammation	[40]
		Diet-related microbial effect on obesity	[48,49]
	+*A. muciniphila*	Impact of microbiota on intestinal inflammation	[50]
		Impact of microbiota on *Salmonella*-induced intestinal inflammation	[51]
SIHUMI	*E. faecalis, R. gnavus, F. prausnitzii, L. plantarum, B. vulgatus, E. coli, B. longum subsp. longum*	Impact of IBD-related microbiota on intestinal inflammation	[52]
	−*E. faecalis*		[53]
SIM	*E. hallii, E. rectale, B. adolescentis, C. aerofaciens, D. piger, R. inulinivorans, R. bromii, B. theta, P. copri, A. muciniphila*	Microbe–diet interaction and impact on metabolism	[41]
MET-1	4× *Bifidobacterium* species, *C. aerofaciens, B. ovatus, P. distasonis,* 2× *Lactobacillus* species, *S. mitis, F. prausnitzii, C. cocleatum, A. intestine, Blautia sp.,* 2× *Dorea* species, *L. pectinoshiza,* 2× *Roseburia* species, 4× *Ruminococcus* species, 7× *Eubacterium* species, *E. coli, Raoultella* sp.	Host–microbe interaction and protection against systemic disease	[54]
	*B. theta*^1^ , *B. vulgatus, B. longum, B. hansenii, C. scindens, C. aerofaciens, E. coli, E. ventriosum, F. prausnitzii, L. rhamnosus*	Host–microbe metabolic cross-talk and impact on metabolism	[42]
	*E. coli* K-12, *L. johnsonii, B. longum, E. coli* Nissle	Microbial cooperation and competition	[45]
	*B. ovatus, B. vulgatos, B. theta* ^1^	Microbial cooperation	[46]
	*B. longum subsp. infantis, B. breve, B. bifidum, B. dentium*	Host–microbe interaction and microbial impact on nervous system	[55]
	*R. gnavus*, *B. theta*, *C. hathewayi*, *C. orbiscindens*	Mechanisms of colonization resistance against *C. perfringens*	[56]
	*C. ramosum, C. asparagiforme, C. indolis, C. hathewayi, C. bolteae, Clostridiales* 1_7_47FAA, 2× *Clostridium* species, *C. scindens, Clostridiaceae* JC13, 3× *Lachnospiraceae* species, *B. producta, E. fissicatena, Ruminococcus sp*. ID8, *A. colihominis*	Impact of microbiota on chronic intestinal inflammation	[57]
	*B. caccae, B. ovatus, B. theta*^1^, *B. uniformis, B. vulgatus, B. cellulosilyticus* WH2, *C. scindens, C. spiroforme, C. aerofaciens, D. longicatena, E. rectale, F. prausnitzii, P. distasonis, R. obeum, R. torques*	Microbe–diet interaction	[43]
		Microbe–microbe interactions	[44]
	−*E. rectale,* −*F. prausnitzii,* −*R. torques*	Microbe–diet interaction	[58]
	*B. caccae, B. ovatus, B. theta* ^1^ *,* *E. rectale, C. aerofaciens, C. symbiosum, E. coli, M. formatexigens*	Microbe–diet interaction and impact on metabolism	[59]
	*+ D. piger, + E. rectale, + B. hydrogenotrophica*		[60]
B4PC2	*B. uniformis, B. vulgatus, B. producta,* *P. distasonis, C. hylemonae, C. hiranonis*	Host–microbe metabolic cross-talk and bile acid metabolism	[61,62]

^1^ Abbreviations: B. theta—B. thetaiotaomicron.
